# Effect of combined application of high- and low-intensity lasers on dentin hypersensitivity: A randomized clinical trial

**DOI:** 10.15171/joddd.2018.008

**Published:** 2018-03-14

**Authors:** Zohreh Tabibzadeh, Reza Fekrazad, Azadeh Esmaeelnejad, Mohammad Mostafa Shadkar, Zahra Khalili Sadrabad, Morteza Ghojazadeh

**Affiliations:** ^1^Department of Periodontics, Faculty of Dentistry, Shahid Beheshti University of Medical Sciences, Tehran, Iran; ^2^Department of periodontology, Dental Faculty-Laser research center in medical sciences, AJA university of medical sciences, Tehran, Iran; ^3^International Network for Photo Medicine and Photo Dynamic Therapy (INPMPDT), Universal Scientific Education and Research Network (USERN), Tehran, Iran; ^4^Department of Periodontics, Faculty of Dentistry, Shahid Beheshti University of Medical Sciences, Tehran, Iran; ^5^Dental Practitioner, Private Practice, Tabriz, Iran; ^6^Department of Pedodontics, Faculty of Dentistry, Tabriz University of Medical Sciences, Tabriz, Iran; ^7^Research Center for Evidence Based Medicine (RCEBM), Tabriz University of Medical Sciences, Tabriz, Iran

**Keywords:** Diode laser, dentin hypersensitivity, desensitizing effect

## Abstract

***Background.*** Diode lasers (DLs) have demonstrated equal or better desensitizing effects than fluoride varnish, 10% potas-sium nitrate (NK) gel and Gluma. The current study evaluated the desensitizing effect of combined application of DLs with two different output powers and compared it with single DL therapy.

***Methods.*** Sixty-two hypersensitive teeth were allocated randomly into two groups: the single group was treated with 3-W DL beam once and in the combined group, the teeth were irradiated three times (the first time with 0.2-W and then with 3-W and the second and third times, 48 and 96 hours after the baseline visit, with 0.2-W DL beams). The amount of dentin hyper-sensitivity (DH) was evaluated, immediately before and after each visit, and 1 week and 1 and 3 months after the first visit. Data analysis was performed using chi-squared test, repeated measurement of ANOVA and Mann-Whitney U test. P<0.05 was considered statistically significant.

***Results.*** Statistically significant changes were observed in the means of VAS indices between all the measurement intervals and pretreatment measures, in both experimental groups (P<0.001). The difference in VAS reduction among the groups was not significant when the hypersensitive teeth were stimulated by a periodontal probe and a jet of air (P=0.63 and P=0.12).

***Conclusion.*** The results of the present study showed that using both high-intensity and combined DL beams gives rise to significant reductions in DH. There was no significant difference between combined and single laser therapies in the treatment of tooth hypersensitivity.

## Introduction

Dentin hypersensitivity (DH) is a frequently occurring annoying condition amongst 30‒40-year-old individuals.^1^ The main symptom of this problem is an acute, sharp and located pain after mechanical, thermal or chemical stimulation.^2-4^ DH usually occurs in the teeth with dentinal tubules exposed to the oral environment and is explained by the theory of hydrodynamic, which introduces dentinal tubule fluid’s movement as a main factor for the stimulation of pulpal receptors.^5^ Therefore, sealing of these exposed tubules can reduce the intensity of pain and discomfort.

Unfortunately, most treatment methods, including the application of potassium ion, oxalates, sodium fluoride and resin bonding agents, are ineffective or produce short-lived desensitizing effects.^6,7^

In mid-1980s, application of laser beams in the treatment of DH was recommended for the first time^8^ and various laser systems have been used to this end. Diode lasers (DL) have been tested in various studies with different output powers and they have demonstrated an equal or better desensitizing effect than fluoride varnish, potassium nitrate gel and Gluma.^9-12^ In another study, cyanoacrylate application results were similar to low-intensity lasers.^13^ Yilmaz et al^14^ reported an equal desensitizing effect for DL and Er,Cr:YSGG lasers, and Dilsiz et al^15^ recommended that Nd:YAG laser is more efficient than DL. Gomi et al^16^ reported a 100% success rate for desensitizing effect of DL and long-term reduction of DH has been shown by Wilder-Smith et al, Pinheiro et al and Marsilio et al.^17-19^ When testing DLs at distinct wavelengths, Almeida Lopes et al^20^ observed that the effect of lasers on cell growth depended on the output power and it was not related to the wavelength. Another investigation revealed that 980-nm **diode** laser can block the dentinal tubules entirely, regardless of the output powers of 2, 3 and 4 Ws.^21^ In a recent in vitro study, when applying a 980-nm diode laser beam with 0.5-, 0.7- and 1-W output powers, **Rizzante** et al demonstrated a greater reduction in dentin hydraulic conductance, as the irradiation power increased.^22^ It seems that lasers with different ranges of output powers affect DH by two different mechanisms: high-power lasers by melting and fusing the peritubular dentin and low-power lasers by antiinflammatory effects and increasing the cellular metabolic activity of odontoblasts.^23-24^ Although the efficiency of DLs in DH management has been reported in various studies,^10-15,25^ prior to the current study, there is no published data on the efficacy of combined application of DLs with two different output powers in order to apply both mechanisms in the treatment of DH. The aim of this study was to evaluate the desensitizing effect of combined application of DLs with two different output powers and compare it with single DL therapy.

## Methods

Eight patients (6 females and 2 males) with a total of 62 hypersensitive teeth, from the maintenance program of the Periodontology Department of Shahid Beheshti Dental School, Tehran, Iran, were enrolled in the study. A power calculation indicated that a minimum of 20 teeth per group were required to detect a significant difference between groups with alpha risk set at 0.05 and beta risk at 0.2. The sample size was increased to 31 teeth per group to compensate for potentially larger number of dropouts. Mean age was 41.7 years (minimum 20, maximum 53) and each patient had at least 4 teeth with hypersensitivity. Shahid Beheshti University Ethics Committee approved the study protocol under the code 3065.

Subjects who had teeth with cracks, restorations in cervical areas, carious lesions and non-vital teeth, those with painful and systemic illnesses and those who were on analgesics or anti-inflammatory medications were excluded. The degree of sensitivity was determined with two thermal and tactile stimuli: a 3-second air blast (60 psi at 22°C) at a distance of 2‒3 mm from the tooth surface and a controlled-pressure probe (AESCULAP 0.2 N, USA) by mesial-to-distal movement on the mid-buccal surface. Visual analog scale (VAS) was used to register the severity of pain (0 = no pain, 10 = severe pain). The hypersensitive teeth in each patient were allocated randomly into two groups: single DL-treated and combined DL-treated. All the teeth were flossed and polished before treatment and cotton rolls were used for isolation. The treatments were carried out with a Diode laser device (Doctor Smile, Lambda SPA, Italy). The first experimental group was treated for 20 seconds with a 3-W DL beam (wavelength=980 nm, 30 Hz, fiber=300 µ, Single Pulse mode) once. The teeth in the second group were irradiated three times in three treatment sessions: In the first session, the teeth were irradiated for 20 seconds with an 0.2-W beam (wavelength=980 nm, fiber=300 µ, continuous wave mode), then for 20 seconds with 3-W output power DL; second and third sessions were 48 and 96 hours after the initial visit, in which the teeth were treated for 20 seconds with 20-Hz and 0.2-W diode laser beam. All the patients were instructed in using a brushing method (modified Bass‏ technique) and were given a standardized toothbrush (Cross Action, Oral B, Germany) and toothpaste (Complete 7, Crest, Germany). The severity of pain was assessed immediately before and after each treatment session and also at 1-week and 1- and 3-month intervals after the first visit. Laser application was carried out by a single investigator and sensitivity assessment was performed by another blind examiner. All the data were presented as Mean ± SD. SPSS 16.0 was used for data analysis. The main statistical assessments were repeated measurement of ANOVA and Mann-Whitney U test. P<0.05 was considered statistically significant.

## Results

Evaluation of the results of repeated measurement of ANOVA indicated statistically significant changes in the means of VAS in all the measurement intervals and pretreatment measures in both study groups (P<0.001). The VAS reduction difference between the two study groups was not statistically significant when a probe was used for assessment (P=0.63). In addition, the difference between the two groups was not significant when an air jet was used (P=0.12).

With the use of a periodontal probe, 68.25±14.5% reduction in mean VAS scores for the combined group and 82.5±11.2% reduction in mean VAS scores for the single group were noticed until the follow-up period ended. The corresponding values were 83.14±6.5% and 69.2±8.02%, respectively, when an air jet was used. The results of Mann-Whitney U test indicated that the differences between these values were not statistically significant (P=0.34, when a probe was used andP=0.062, when an air jet was used) (Tables 1 and 2). When a probe was used to stimulate the teeth by a probe, reductions in DH were observed in 34.4% of the irradiated teeth in the combined-DL group and 51.5% of treated teeth in the single-DL group, until 3 months of follow-up period ended. This difference was not statistically significant (P=0.26). When stimulation was carried out by a jet of air, the corresponding values were 87.5% and 72.7%, respectively, with no statistically significant differences (P=0.26).

**Figure 1:**
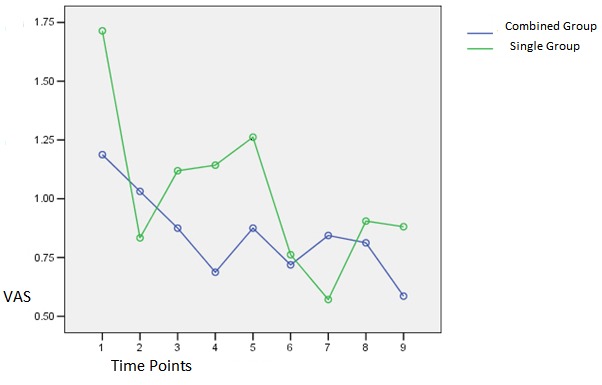
VAS index changes in the two groups at different time intervals when a periodontal probe was used.

**Figure 2:**
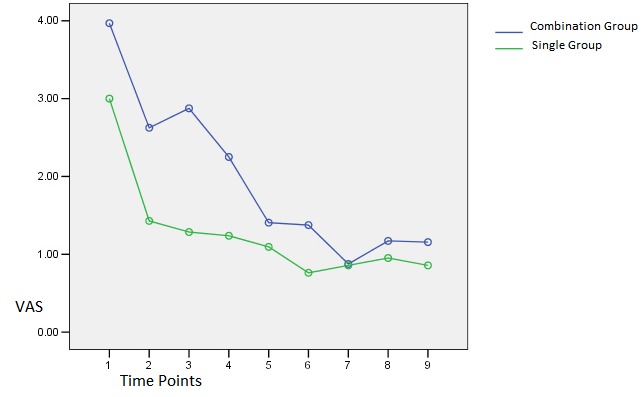
VAS index changes in the two groups at different time intervals when an air jet was used.

Figures 1 and 2 show VAS index changes in the two groups and at different times when the teeth were stimulated by a probe and a jet of air.

None of the patients showed adverse effects after laser therapy.

**Table 1:** Mean VAS scores in the two groups at different time intervals, with the use of a periodontal probe for stimulation

	**Combined Group**	**Single Group**	**P**
**Session 1**			
BT	1.18‏±0.36	1.66‏±0.39	0.12
AT	1.03‏±0.28	0.76±0.20	0.70
**Session 2**			
BT	0.87‏±0.25	0.97±0.22	0.38
AT	0.68±0.23	1.08±0.26	0.10
**Session 3**			
BT	0.87±0. 27	1.14±0.29	0.28
AT	0.71±0.27	0.76±0.21	0.36
1 week	0.84±0.26	0.54±0.15	0.63
1 month	0.81±0.29	0.66±0.17	0.70
3 months	0.58±0.25	0.77±0.18	0.23

BT: before treatment; AT: after treatment Session 1: initial visit; Session 2: 48 hours after initial visit; Session 3: 96 hours after initial visit

**Table 2:** Mean VAS scores in the two groups at different time intervals, with the use of an air jet for stimulation

	**Combined Group**	**Single Group**	**P**
**Session 1**			
BT	3.96‏±0.53	3.69‏±0.53	0.69
AT	2.62‏±0.45	2.48‏±0.59	0.45
**Session 2**			
BT	2.87‏±0.48	1.20‏±.35	0.01
AT	2.25±0.46	1.16±0.30	0.17
**Session 3**			
BT	1.04±0.29	0.95±0.30	0.19
AT	1.37±0.28	0.76±0.29	0.10
1 week	0.87±0.24	1.66±0.42	0.32
1 month	1.17±0.24	1.54±0.40	0.91
3 months	1.15±0.34	1.33±0.29	0.44

BT: before treatment; AT: after treatment Session 1: initial visit; Session 2: 48 hours after initial visit; Session 3: 96 hours after initial visit

## Discussion

After invention of the first laser in 1960 by Maiman, several studies were designed to examine the ability of various lasers in blocking dentinal tubules, followed by reduction or complete resolution of the symptoms of hypersensitive teeth. These studies showed different results because of the difference in laser parameters such as the laser type, power, radiation frequency, exposure time, exposure mode etc.^26^ Diode or Gal-Al-As laser was used in the present study. It is a semiconductor laser whose different wavelengths have been used to treat hypersensitive teeth (780, 900 and 810 nm)^27^ and various studies have showed its efficiency in the treatment of DH.^10-14^

Use of a pressure-controlled probe with a modified sharp tip, designing the study as a clinical evaluation, presence of two study groups in each patient and blindness of patients and the observer to the type of treatment method of each group, are some of the advantages of the present study. DL system was used in the study because it is inexpensive, lightweight and available.

The body of science indicates that the function mechanisms of high and low intensity lasers on dentin hypersensitivity reduction are different. It seems each laser group has its unique way to affect tooth structure. This study was designed to use these two completely different mechanisms simultaneously to maximize the effect of lasers on reducing hypersensitivity.

According to the data obtained from the current study, application of high-intensity lasers (808-nm diode with 3-W output power) reduces DH. The mean reduction amounts after three months, in high-intensity group, with the use of thermal and mechanical stimuli, were 69.2±8.02% and 82.5±11.2%, respectively.

High-intensity lasers reduce DH in various ways, the most important of which is photo-thermal effect that melts and fuses the hard tissue with the smear layer and occludes tubules.^26,27^ Moreover, the laser energy may lead to the necrosis of sensory terminals at the orifice of dentinal tubules.^26^

Stabholz et al^26^ reported dentin permeability reduction and melting of dentin surface when they used high-intensity laser (Nd:YAG) with 3-W output power. They also observed that Nd:YAG laser affects DH through the occlusion of dentinal tubules. Other researchers such as Lan et al^28^ in 1996, Glauche et al^29^ and Gholami et al^27^ in 2011 reported similar results in microscopic studies. However, Whitters et al^30^ introduced the direct analgesic action of Nd:YAG laser as the only possible mechanism of pain relief. Orchardson et al^6^ claimed that the Nd:YAG laser effect on DH is due to depolarization inhibition of C and Aδ fibers.

Botzenhart et al^31^ showed in an in vitro investigation that application of 809-nm DL beam with 1-W output power, 10-Hz frequency and 60-second exposure time cannot produce predictable microscopic changes on dentin and probably it results in hypersensitivity reduction due to the direct effect on the pulp. However, Gholami et al^27^ reported that DL beam with 2-W output power can seal dentinal tubules to a low degree and probably has a desensitizing effect. Consistent with his results, Umana et al^32^ showed that diode laser irradiations at 0.8 and 1 W led to occlusion of dentinal tubules. Furthermore, Kimura et al^7^ in 2000 and Goharkhay et al^33^ in 2007 demonstrated that DL can partially block the dentinal tubules. Moreover, it has a direct analgesic effect.

In 2002, Schwarz et al^34^ reported a 50% reduction in DH 6 months after application of a high-intensity Er:YAG laser beam (80 mj/pulse, 3 Hz). They concluded that DH resolution with the use of Er:YAG laser is more effective than dentin protector and similar to the results of the current study, indicating the high-intensity laser effectiveness; the reason for differences between its effectiveness in two studies can be attributed to differences in laser types, wavelengths, intensity and other radiation properties.

Ciaramicoli et al^35^ and Gutknecht et al^36^ applied Nd:YAG lasers and reported 80% and 94.5‒98.6% reductions in DH, respectively. These results clearly show the efficacy of high-intensity Nd:YAG laser beams.

In our study, VAS reduction was noticed between the beginning and termination of each visit in both groups. As we mentioned previously, the treatment was carried out only during the first visit in the high-intensity laser group. Therefore, the reduction observed between the baseline and postoperative interval in the second and third visits might be the result of the placebo effect of laser used in the teeth of the combined group in the oral cavity of the patient in the same visit.

In previous studies, no one has used the combination of high- and low-intensity lasers for hypersensitivity reduction; therefore, we decided to apply this treatment approach to benefit from the two different effects of high- and low-intensity lasers.

According to the results of the current study, use of a combination of high- and low-intensity lasers can result in a significant decrease in DH. The mean hypersensitivity reductions after 3 months, in the combination group, when using thermal and mechanical stimuli, were 83.14±6.5% and 68.25±14.5%, respectively.

The function of low-intensity lasers is attributed to their direct effect on the pulp tissue. This therapeutic effect can bring about analgesic and antiinflammatory results and is related to the lasers which have high penetration depth, such as 810 nm with DL and Nd:YAG. These lasers affect pulp tissue components and induce analgesia. Furthermore, the photo-modulating effect, stimulation of odontoblasts in dentin‒pulp complex and also formation of tertiary dentin are some of the other reasons of DH reduction over a long period of time.^1,27^

Senda et al^37^ was one of the first researchers who used low-intensity lasers (He-Ne) in the treatment of DH. They applied the output power of 6 mW which does not cause morphologic changes on the surface of enamel or dentin and reported that the effect of this treatment was from 2.5% to 100%, claiming that use of He-Ne laser has more effects on action potential than Aδ or C fibers.

In another study, Wakabayashi et al^24^ reported that DL blocks depolarization of C fibers and some other researchers concluded that this laser can stimulate the normal physiologic cellular function.^8,23^

Sicilia et al^10^ in 2009 evaluated the effect of low-intensity DL (810 nm, 1.5‒2.5 mW, 1 minute) and reported that it causes 65.7% and 92% reductions in hypersensitivity caused by thermal and mechanical stimuli, respectively. This study showed efficacy of DL in hypersensitivity treatment and the differences in hypersensitivity reductions between that study and the present study can be attributed to differences in radiation properties and study durations.

Pesevesca et al^9^ in another study used low-intensity DL (630‒670 nm, 100 mW/cm²) and reported complete resolution of hypersensitivity in 86.6% of patients 4 days after treatment. They introduced low-intensity lasers as a successful treatment method. Radiation properties, duration of the study and use of only one stimulant type are some of the differences between that study and the present one. The short duration of this study decreases the validity of the results.

Dilsiz et al^15^ in 2009 showed that 685-nm DL beam significantly reduced hypersensitivity after 2 months. Also, Ladalardo et al^1^ claimed that both 830-nm and 660-nm DL beams caused hypersensitivity reduction. Both these studies show the efficacy of this type of laser therapy and confirm the results of the present study. In another research, Matsumoto et al^38^ reported 85‒100% pain relief with the use of DL (30 mW, 0.5‒3 minutes). Gerschman et al^8^ used DL beams with 30-mW output power and reported 65‒67% of hypersensitivity reduction. Marsilio et al^19^ observed 87% pain relief with the application of DL beams with 15-mW output power. The effects of GaAlAs laser in Kimuraet al.^7^ study were 30‒100% and 73.3‒100% when 20‒100-mW and 2.4-mW output powers were used, respectively.

Wakabayashi et al^24^ also reported 98% pain reduction after application of 780-nm DL beam with output power of 30 mW. Tissue response after laser radiation is affected by factors such as wavelength, output power, radiation mode and radiation dose.^18^ This can be the reason for differences in the reported values.

According to the results of our study, there was no significant difference between the two study groups in DH reduction. Using combined laser therapy has its own limitations. It increases the chair time and the clinical procedure is more complex than single laser therapy. The cost-benefit should thus be evaluated when deciding to choose a special treatment approach.

In the present study, after evaluating the results, we concluded that although the effect of high-intensity laser with mechanical stimuli was more than the effect of combined application of lasers (the difference was not statistically significant), the consistency of the treatment was significantly higher in the combination group. In addition, the effect of combination laser therapy was more than high-intensity laser application, when using thermal stimuli (although there was no significant difference). The reason might be attributed to the low-intensity laser effect in the combination group, in stimulation of odontoblasts and formation of tertiary dentin.

The definite mechanism of dentin hypersensitivity is not clear yet and some other theories, other than hydrodynamic theory, have been proposed. This may be the reason for differences between the results when thermal and mechanical stimuli are used.

The two therapeutic systems of low-intensity and high-intensity lasers affect the two sides of dentinal tubules. The pulpal effects of low-intensity lasers, when used alone, are probably more reversible because of continuous stimulation from the outside. External blockage of dentinal tubules can also be eliminated because of abrasion and the presence of microorganisms. The reason for the persistence of treatment results in the combination group might be attributed to the effect of laser on both sides of dentinal tubules.

As the combined application of lasers with different intensities is a new approach in DH treatment, various other studies are needed to assess its efficacy and compare it with the single high-intensity laser method.

## Conclusion

According to the results of the current study, it was concluded that using high-intensity and combined DL beams gives rise to significant reductions in DH. There was no significant difference between the combined and single laser therapies in the treatment of tooth hypersensitivity.

## Acknowledgments

The manuscript is totally driven from a thesis which was performed under the supervision of Dr. Zohreh Tabibzadeh and Dr. Reza Fekrazad in Shahid Beheshti Faculty of Dentistry.

## Funding

The Research Center of Shahid Beheshti University of Medical Sciences supported the study.

## Competing interests

The authors declare that they have no competing interests with regards to authorship and/or publication of this paper.

## Ethics approval

The study was approved by the University of Shahid Beheshti Ethics Committee under the code 3065.
